# Mechanical Behavior of Stainless Steel Fiber-Reinforced Composites Exposed to Accelerated Corrosion

**DOI:** 10.3390/ma10070772

**Published:** 2017-07-08

**Authors:** Caitlin O’Brien, Amanda McBride, Arash E. Zaghi, Kelly A. Burke, Alex Hill

**Affiliations:** 1 Civil and Environmental Engineering Department, University of Connecticut, 261 Glenbrook Road, Unit 3037, Storrs, CT 06269-3037, USA; 2 Chemical and Biomolecular Engineering, University of Connecticut, 191 Auditorium Road, Unit 3222, Storrs, CT 06269-3222, USA; 3 Polymer Program, Institute of Materials Science, University of Connecticut, 97 North Eagleville Road, Unit 3136, Storrs, CT 06269-3136, USA; 4 Biomedical Engineering, University of Connecticut, 260 Glenbrook Road, Unit 3247, Storrs, CT 06269-3247, USA; 5 Chemical Engineering, Northeastern University, 360 Huntington Ave., Boston, MA 02115, USA; hill.al@husky.neu.edu

**Keywords:** composite, digital image correlation (DIC), metal fiber-reinforced polymer, mechanical properties, pitting corrosion, stainless steel

## Abstract

Recent advancements in metal fibers have introduced a promising new type of stainless steel fiber with high stiffness, high failure strain, and a thickness < 100 μm (<0.00394 in.) that can be utilized in a steel fiber-reinforced polymer. However, stainless steel is known to be susceptible to pitting corrosion. The main goal of this study is to compare the impact of corrosion on the mechanical properties of steel fiber-reinforced composites with those of conventional types of stainless steel. By providing experimental evidences, this study may promote the application of steel fiber-reinforced composite as a viable alternative to conventional metals. Samples of steel fiber-reinforced polymer and four different types of stainless steel were subjected to 144 and 288 h of corrosion in ferric chloride solution to simulate accelerated corrosion conditions. The weight losses due to corrosion were recorded. The corroded and control samples were tested under monotonic tensile loading to measure the ultimate stresses and strains. The effect of corrosion on the mechanical properties of the different materials was evaluated. The digital image correlation (DIC) technique was used to investigate the failure mechanism of the corrosion-damaged specimens. Overall, steel fiber-reinforced composites had the greatest corrosion resistance.

## 1. Introduction

Incorporation of metal fibers into conventional fiber-reinforced polymer (FRP) composites creates a strong, ductile, hybrid fiber composite that reduces material cost and weight in comparison to metals [1,2]. FRP composites are most commonly comprised of glass or carbon fibers set in polymer resin, and utilized in corrosive environments to construct tanks, piping, scrubbers, beams, grating, and other components [3]. Their superior durability has been widely studied for the repair and retrofit of corrosion-damaged structural elements [4,5,6,7,8,9]. Though FRP composites show promise for application in various industries, the brittle nature of the fibers and limited capacity for energy absorption restrict the ability of the FRP to perform in critical structural elements that need protection from extreme events. Studies have investigated ways to improve the ductility of composites, and the incorporation of metal fibers into FRP composites for this purpose has been recently studied [10,11,12,13,14,15]. Metal fibers have high stiffness, high strength, ductile failure, high tensile energy absorption, high electrical conductivity, and superior structural integrity following impact [16]. Recent advancements in the manufacturing of metal fibers have introduced a promising new type of stainless steel fiber with high stiffness, high failure strain, and a thickness < 100 μm (<0.00394 in.). The tensile and impact performance of a unidirectional (UD) and cross-ply polymer composite utilizing these fibers has been recently investigated. The effect of the combination of brittle and ductile matrices, fiber architecture, and modifying adhesion between fiber and matrix has also been explored [10,11,12,13,14,15,17,18]. However, while FRP composites offer excellent corrosion resistance when the optimal fiber and resin is utilized [3,19,20], the addition of steel fiber reinforcement may decrease this durability. Results demonstrate the potential of steel fibers to improve the failure strain and energy dissipation performance of composites [1,2], but little has been done to study the corrosion resistance of FRP–stainless steel fiber-reinforced polymer (SFRP) composites [4,5,6,7,8,9].

Steel performs poorly in corrosion settings as it rusts in air, corrodes in acids, and scales at higher temperatures [21]. To increase corrosion resistance in steel, iron-chromium, nickel, and other alloying metallic elements are added during production to create stainless steel [22]. Once stainless steel is in contact with oxygen, a protective chromium oxide layer forms on its surface that acts as a passive film and enables the material to self-repair [23]. Stainless steels are separated into different groups based on the alloys added and their microstructure [21,23]. Each group always contains varying percentages of chromium, nickel, and carbon, as well as additional alloys. As discussed, chromium forms the passive film of chromium oxide, making the steel corrosion resistant. The presence of a minimum of 10.5% chromium gives stainless steel its corrosion resistance property [21,23,24]. Nickel increases ductility and toughness, as well as increasing its strength and corrosion resistance in high temperatures [21,24]. Carbon is used in small percentages; while it strengthens stainless steel, it decreases toughness and promotes the formation of precipitates that hinder corrosion resistance [21,24]. Small percentages of additional elements, such as copper, columbium, aluminum, and molybdenum, are added to achieve desirable characteristics [21,24]. The stainless steel groups of particular interest in this study include ferritic, austenitic, and precipitation-hardening (PH) stainless steels. Ferritic stainless steels are resistant to corrosion and scaling at elevated temperatures. They are magnetic and non-hardenable. Austenitic stainless steels are the most commonly produced grades because of their formidability and corrosion resistance. Precipitation-hardening (PH) stainless steels are known for developing high strength and hardness through heat treatment [24].

The ability of stainless steel to resist surface corrosion and maintain strength at high temperatures, as well as its ease of maintenance, allows it to be utilized in diverse applications. Stainless steel is commonly used in consumer products and in equipment for the oil and gas industries; it also has applications in the chemical process industry and the food and beverage industry [21].

Despite their resistance to general surface corrosion, stainless steels are susceptible to pitting corrosion, a highly localized type of corrosion [22]. Pitting corrosion is an electrochemical oxidation-reduction process that occurs in localized areas of the passive film on the surface of stainless steel [22]. Pits are small cavities or holes and can occur in many types of atmosphere exposure but usually the environments contain chlorides or sulfides [23,25]. Once this passive film breaks down, metastable pitting begins. If the area cannot repassivate, the metastable pitting transitions to a stable pit [26]. Depassivation of the film causes the affected area to become anodic, while the area around it is cathodic. This increase in metal ions and electrons creates current and an electrical potential difference between the anodic zone and the cathodic zone. This potential, known as the pitting potential, gives one a way to measure the ability of a grade of stainless steel to resist pitting. The pitting potential is the potential needed to initiate pitting. Therefore, if the potential of a stainless steel in a given medium is higher than its respective pitting potential, pitting corrosion occurs [23].

The pitting potential, however, is difficult to determine due to numerous kinetic effects [27]. Furthermore, the pitting potential is unreliable, as many investigations have found pitting corrosion to occur before reaching the pitting potential [28]. Pitting corrosion is a sporadic and stochastic process, making it difficult to predict the likelihood of this common, and catastrophic, form of failure to occur in stainless steel structures [22]. Although corrosion pitting can cause little overall metal loss, even a single perforation can create failure or stress corrosion cracks which reduce the life cycle of the stainless steel [25]. Additionally, though pits appear small on the surface, there can be a larger pit cross-sectional area deeper inside the metal. This often makes pitting corrosion attacks undetectable until a perforation or leak occurs [22].

Countless engineering failures have occurred in recent years due to corrosion. A 2016 study estimated that 42% of failures in engineering components are due to corrosion. This is the dominant cause of failure and includes general corrosion, pitting corrosion, corrosion in gaps, stress corrosion cracking, and corrosion fatigue [29]. The potential for catastrophic failure due to corrosion has encouraged scientists to conduct numerous studies to further understand pitting corrosion in stainless steel [26,30], factors that affect stainless steel corrosion [26,31,32,33,34,35], and how to control and prevent pitting [36,37,38]. It is known that corrosion is affected by pH, temperature, concentration of corrosive material, exposure time, and surface finish. Studies have concluded that the rate of corrosion increases along with increases in pH, increases in temperature [31,32], and increases in concentration of chloride [32,34,35]. A study by Liebhafsky and Newkirk, in which AISI Type 302 stainless steel with different surface conditions are submerged in ferric chloride, found that dissolution of the samples can approach completion with little visual alteration in appearance [35]. This lack of visual confirmation of dissolution is a concerning discovery. A study by Burnstein and Pistorius found that Type 304 samples with a smoother surface finish had less frequent metastable pitting sites when submerged in a chloride solution. However, the probability of achieving stable pits was greater if the surface of the sample was smoother [33].

The corrosive properties of fiber composites have been researched in numerous studies. Stress corrosion cracking (SCC) is of main concern in composites and has been studied extensively [3,39,40,41,42,43,44,45,46,47,48,49]. Stress corrosion cracking occurs from the combination of a corrosive environment and mechanical stress. The progression of SCC can rapidly progress and result in sudden failure of composites while in service [48]. The main mode of failure of composites in acidic environments is fiber degradation [49].

Research has identified critical factors when considering composites in corrosive environments. They include resin and fiber type, type of corrosive environment and concentration of corrosive materials, composite surface conditions, external stress, and micro crack size [3,48]. Resin must be corrosion resistant. Resin can also modify the stress acting on fibers, and this can control the rate of crack growth during stress corrosion [48]. Megel et al. concluded that an electrical grade glass (E-glass) composite with vinyl ester resin is approximately ten times more resistant to the initiation of SCC than an E-glass composite with epoxy resin when subjected to nitric acid. E-glass/epoxy composites exhibited approximately 5 times higher resistance to the initiation of SCC than an E-glass/modified polyester composite [43]. Kumosa et al. and Kumosa et al. reported similar conclusions [40,42]. Composites with electrical/chemical resistance glass (ECR-glass) fiber reinforcement have been proven superior to E-glass fiber reinforcement when considering stress corrosion in nitric acid [41,45]. Acid-resistant fiber types and resin gel-coats have also been considered a valuable option when subjecting composites to corrosive environments [44]. A study by Dai et al. concluded that when considering FRP rods, the stress corrosion fracture time of the FRP rod decreases with increase of the micro-crack depth and the circumferential angle of the micro-crack on the surface of the rod [39]. Studies by Wei et al. and Huang Gu found that E-glass fibers’ tensile strength decreases with increased acid treatment time when subjected to sodium hydroxide treatments [46,47].

Few studies exist researching the properties of stainless steel fiber composites in corrosive environments; therefore, how the corrosion of composites consisting of stainless steel fibers embedded in a polymer matrix compares to bulk steel materials (e.g., steel plates) is not known. Additionally, steel fibers are inherently more vulnerable to corrosion than traditional stainless steel plates, as the small diameter of the fibers results in a much higher surface area of steel reinforcement that is susceptible to corrosion. Embedding the steel fibers in resin is hypothesized to reduce this vulnerability to permit the steel composite to be resistant enough to be used on its own or in combination with fiberglass in a hybrid composite. The focus of this study is to investigate the corrosive properties of a SFRP composite and compare them with stainless steel plates of different types.

## 2. Materials and Methods

### 2.1. Overview

The main goal of this study is to determine the impact of corrosion on mechanical properties of steel fiber-reinforced composites and to compare the corrosion of composite materials with plates of different types of stainless steel. This experimental study investigates the corrosion resistance of these steel-reinforced composite specimens, which will be important to evaluate if the composites are a viable alternative to conventional metals for resistance to corrosive environments. Samples of steel fiber-reinforced polymer (SFRP) and four different types of stainless steel were subjected to 144 and 288 h of corrosion in ferric chloride solution according to ASTM G48-11 [50]. The weight losses of the materials due to corrosion were recorded. The corroded and control samples were tested under monotonic tensile loading to measure the ultimate stresses and strains. The effect of corrosion on mechanical properties of the different materials was evaluated. The digital image correlation (DIC) technique was used to investigate the failure mechanism of the corrosion-damaged specimens.

### 2.2. Materials

#### 2.2.1. Stainless Steel Samples

**Figure 1:**
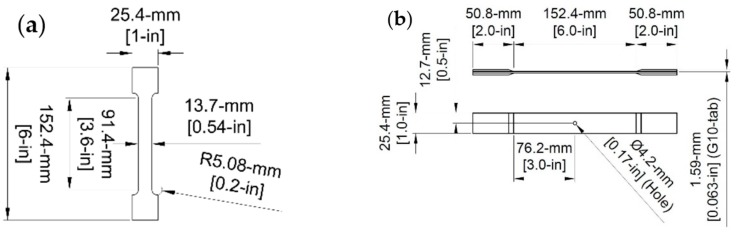
(**a**) Stainless steel dog-bone shape; and (**b**) Composite coupon dimensions with end tabs.

Four different stainless steel samples were selected for this study: AISI Type 430 (UNS S43000), AISI Type 630 or 17-4 PH (UNS S17400), AISI Type 316 (UNS S31600), and AISI Type 304 (UNS S30400). These types are widely used because of their superior corrosion resistance. Four dog-bone shaped samples of each steel type were manufactured with a gage length of 76.2 mm (3 in.) and gage width of 13.7 mm (0.54 in.). A dog-bone shape was used to confine the failure of the specimens to within the gage length during tensile testing. Geometry of the dog-bone shape is shown in Figure 1a. Type 430 had a thickness of 0.78 mm (0.031 in.), Type 17-4 had a thickness of 0.93 mm (0.038 in.), Type 316 had a thickness of 0.81 mm (0.032 in.), and Type 304 had a thickness of 0.88 mm (0.035 in.). Naming convention of each sample starts with the stainless steel type, followed by a 0, 144, or 288 for specimens exposed to 0 h (control), 144 h, and 288 h of corrosion, respectively. Numbers 1, 2, 3, or 4 at the end indicate a different sample of the same type. For example, sample 17-4-144-2 is one of the Type 17-4 steel samples that was corroded for 144 h.

As discussed, each type of stainless steel consists of different percentages of chromium, nickel, and carbon, as well as other elements. Type 430 is a general purpose ferritic stainless steel that is non-hardenable with good formidability characteristics. It is commonly used in appliances, food equipment, flue liners, and various automotive parts. Type 430 is corrosion resistant in various environments including nitric acid and some organic acids, but is not as resistant to pitting corrosion as other chromium-nickel stainless steels. It reaches optimal corrosion resistance when highly polished or buffed. Like all ferritic grades, Type 430 is highly resistant to stress corrosion cracking [51].

Like all PH stainless steel, Type 17-4 is able to gain high strength and high hardness through heat treatment. This chromium-nickel-copper stainless steel is typically used in aerospace and petrochemical applications and other applications that require high strength and a moderate level of corrosion resistance. It has adequate corrosion resistance comparable to Type 304 or 430 when subjected to atmospheric corrosion or corrosion in diluted acid salts. Stainless steel 17-4 is known to withstand corrosive attacks better than any standard hardenable stainless steel, but can be subjected to crevice or pitting corrosion if exposed to stagnant sea water for any length of time [51].

Type 316 is an austenitic stainless steel typically used in heat exchangers, chemical equipment, and marine applications. Type 316 has excellent tensile, creep, and stress-rupture strengths at elevated temperatures, as well as outstanding formability and weldability. It has higher nickel (10–14%) and molybdenum (2–3%) content than other types in its grade; which allows it to have higher corrosion resistance than Type 304, especially in pitting corrosion attack in chloride environments [51].

Type 304, another austenitic stainless steel, is a multipurpose stainless steel used in food equipment, tubing, and architectural trim. It is one of the most versatile and widely used stainless steels on the market due to its ease of fabrication and outstanding formability and weldability [51]. Its chromium content (typically 17.5–24%) enables it to be corrosion resistant in oxidizing environments. Its nickel content (typically 8–15%) enables it to be resistant to moderately aggressive organic acids [51].

#### 2.2.2. Composite Samples

SFRP samples were manufactured in the form of flat plates using a hand layup method with a thermosetting vinyl ester resin. The plates were heat-cured under compression. The composite samples are named according to the same conventions as the steel dog bone samples, however, the word “Composite” is used. For example, Composite-0-1 denotes the first control composite specimen.

##### Steel Reinforcement

**Figure 2:**
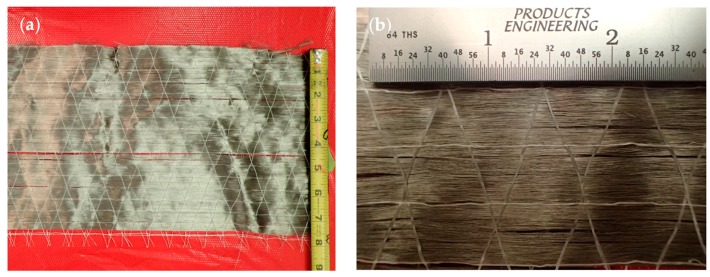
(**a**) Steel reinforcement fibers on the roll; and (**b**) Close up of weave of steel reinforcement fibers.

The steel reinforcement used to manufacture the composite samples is a quasi-unidirectional (UD) stainless steel fiber weave provided by NV Bekaert SA (Kortrijk, Belgium) [52]. The weave has an areal density of 570 g/m^2^ (0.116 lb/ft^2^), and the fibers are a Type 316 stainless steel alloy with a diameter of 30 μm (0.0011811 in.). The roll of fiber weave is shown in Figure 2a and a close-up image of the weave is shown in Figure 2b. Polyethylene succinate (PES) cross yarns with an average diameter of 15 μm (0.000590 in.) maintain the integrity of the weave in the warp (0°) direction without contributing significantly to the mechanical properties of the fabric. The fiber manufacturing process uses a bundle drawing technique, a method that was first used to lower the cost of individually drawing fine wires. This technique draws a number of wires together at the same time and subjects the bundle to a series of drawing and annealing steps. First, the metal is drawn to a diameter of approximately 1 mm (0.0394 in.). Then, the wire is bundled and coated in another metal that can withstand the drawing and annealing processes. The drawing and annealing process occurs until a desired diameter is reached, then the bundles are leached to dissolve the coating material, which releases the individual metal fibers [53]. Annealing the fibers at >800 °C (1472 °F) ensures high strain-to-failure without compromising the stiffness of the fibers. The manufacturer provided the Young’s modulus of the fiber as 193 GPa (28,000 ksi). This particular fiber fabric was first manufactured for research purposes and has limited industry application.

The chemical composition of the stainless steel fibers is presented in Table 1. To determine the chemical composition of the stainless steel, Inductively Coupled Plasma (ICP) spectrometry was implemented. During this process, a small portion of the steel fibers was heated to a plasma state, which emits light. This light is separated into discrete component wavelengths using diffraction grating. The composition of the steel fibers (Table 1) is determined by comparing these component wavelengths to the known distinct emission wavelengths of different elements.

##### Resin

The thermosetting matrix system used to manufacture the SFRP samples is DERAKANE 411-350 (bisphenol A-Type) epoxy vinyl ester resin supplied by Ashland (Covington, KY, USA). It was mixed with manufacturer-recommended ratios of an initiator, methyl ethyl ketone peroxide (MEKP), a promoter, cobalt naphthenate (CoNap 6%), and an accelerator, dimethylaniline (DMA), to obtain optimal polymer cross-linking. DERAKANE resins have become widely used in industry because of their wide range of end-use applications, high resistance to a broad range of chemicals, and ability to hold up in corrosive environments [54]. In structural applications, thermosets are preferred over thermoplastic resins because of their creep resistance over a wide range of temperatures [55].

To characterize stress-strain properties of the resin alone, monotonic tensile testing per the American Society for Testing and Materials (ASTM) standard D638 [56] was performed on dog-bone shaped samples with a 50.8-mm (2-in.) gage length and a 6.35-mm (0.25-in.) width. The Young’s modulus, ultimate tensile strength, and failure strain were found to be 798 MPa (116 ksi), 38.6 MPa (5.6 ksi), and 1.51%, respectively. To investigate the behavior of the vinyl ester after corrosion, two samples were included in the 144-h corrosion bath.

##### Manufacturing of Composite Specimens

**Figure 3:**
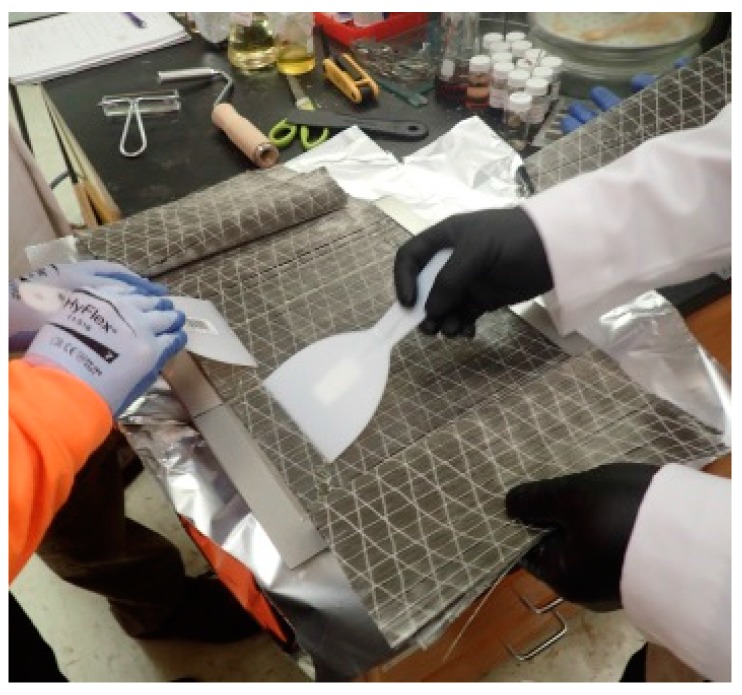
Saturating fibers in epoxy vinyl ester during composite preparation.

A compression molding technique was used to manufacture the SFRP specimens. Care was taken throughout the entire manufacturing process to minimize voids. Eight layers of steel reinforcement were oriented in the longitudinal direction on a 254-mm × 254-mm (10-in. × 10-in.) square steel plate in a 1.8-mm (0.07-in.) thick frame. The reinforcement was saturated in epoxy vinyl ester (Figure 3).

As per the manufacturer’s recommendation, curing of the composite plates was completed at room temperature (21 °C (69 °F)) as well as under a pressure of 7 bar (100 psi) to allow excess resin to bleed out while reaching desired thickness. Following curing, the composite plates were cooled for 30 min under atmospheric pressure and then cut into 25.4-mm (1-in.) wide coupons.

Fiber volume fraction (*v_f_*) was calculated using Equation (1) and is based on the composite thickness (*t*), material density (*ρ*), number of fiber layers (*n*), and fabric area density (*A*). The total fiber volume fraction of the composite was 36.2 ± 0.5%.

*v_f_* = (*n × A*)/(*ρ × t*), (1)

To avoid premature failure due to stress concentrations in the testing grips, 50.8-mm (2-in) wide tabs were applied to the ends of the coupons. The end tabs were G10 fiberglass, an industrial laminate made from glass fabric embedded in epoxy resin. The G10 was beveled and applied to the ends of the samples with a Loctite Armstrong A-12 epoxy adhesive resin [57]. Following the application of the end tabs, the gage length of the composites was 152.4 mm (6 in.). To ensure failure occurred within the extensometer gage length, a hole was drilled in each of the composite samples. Per ASTM specifications, the hole drilled in the center of each specimen had a diameter equal to 1/6 of the coupon width. Overall geometry of the composite specimens with end tabs is shown in Figure 1b.

### 2.3. Experimental Methodology

#### 2.3.1. Application of Corrosion Damage

**Figure 4:**
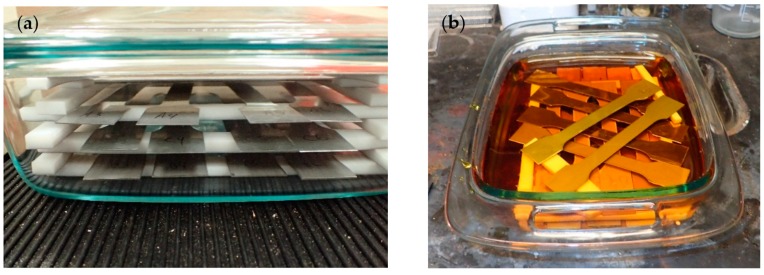
(**a**) Stacking sequence of the specimens; and (**b**) Submersion of the stacked specimens.

The samples were corroded using Method A of the ASTM standard G48-11: Standard Methods for Pitting and Crevice Corrosion Resistance of Stainless Steels and Related Alloys by Use of Ferric Chloride Solution [50]. This method is used for comparing the resistance of stainless steels and related alloys to the initiation of pitting corrosion. A ferric chloride hexahydrate solution was prepared by dissolving 100 g reagent grade ferric chloride (FeCl_3_·6H_2_O) in 900 mL of Type IV reagent water. The test specimens were submerged in the solution in a Pyrex (glass) container and covered. To provide even exposure on the entire surface area and prevent accelerated corrosion from occurring, specimens were stacked on strips of polytetrafluoroethylene (PTFE), a material that is highly compatible with ferric chloride, and it was ensured that none of the samples were touching each other (Figure 4a). Samples were submerged (Figure 4b) for a total of 144 h or 288 h. Typically, corrosion experiments occur in 72-h and 144-h timeframes. Visual observation after 72 h indicated insignificant pitting in some of the samples; therefore, samples were submerged for 144- and 288-h timeframes. After removing the submerged samples, they were rinsed with water and scrubbed with a nylon brush to remove the corrosion product. They were dipped in acetone and allowed to air-dry. The weight of each sample was recorded before and after submersion. Subsequent pitting was documented. The corrosion experiment was conducted at a constant ambient room temperature of 22 °C (71.6 °F).

#### 2.3.2. Tensile Testing

**Figure 5:**
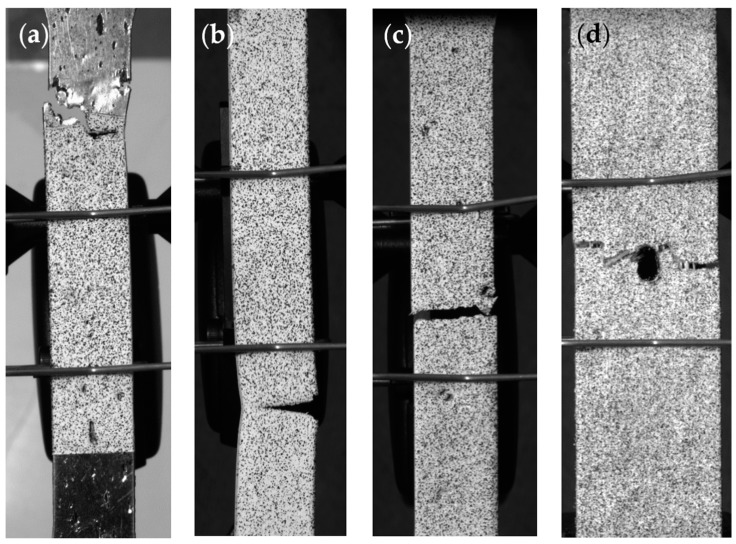
Examples of failures of stainless steel plates due to corrosion including (**a**) above, (**b**) below, and (**c**) within the gage length. (**d**) All composite samples failed within the hole per ASTM standard.

Monotonic tensile testing was performed on each steel specimen to obtain the maximum tensile strength and maximum strain. Monolithic open-hole testing (OHT) was performed on the SFRP composites according to ASTM D5766 [58], and stresses were calculated by dividing forces by the gross cross-sectional area, disregarding the reduced area due to the hole. Loading was in the direction of the fibers. All testing was performed using an Instron (Norwood, MA, USA) 5869 electromechanical universal testing frame with a maximum load capacity of 50 kN (11240 lb). The displacement was applied at 3.81 mm/min (0.15 in./min) until failure. The longitudinal strain was measured using an Instron static axial clip-on extensometer with a 25.4-mm (1-in.) gage length. Strain distributions were measured by a Digital Image Correlation (DIC) system that requires a speckle pattern applied to the face of each sample using a textured spray paint. DIC image data was recorded at a rate of 2 frames per second (fps) using two Point Grey (Richmond, BC, Canada) Grasshopper3 50S5M-C USB3 cameras. In coupon specimens, failure occurred at various locations depending on the pattern of pitting. Thus, for some of the samples, failure did not occur within the gage length. Examples of different failures that occurred in the stainless steel plates are shown in Figure 5a–c. All composite samples failed at the hole, per ASTM standard as shown in Figure 5d.

## 3. Results

### 3.1. Corrosion Damage Measurement

**Figure 6:**
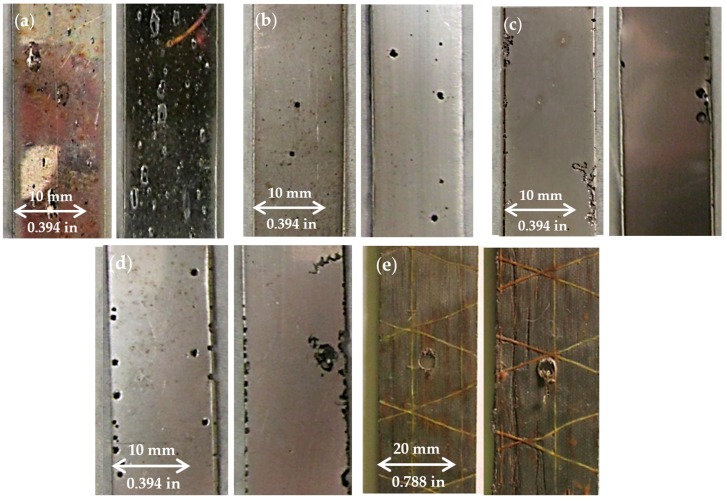
Examples of pitting shape and frequency for (**a**) Type 430, (**b**) Type 17-4, (**c**) Type 316, (**d**) Type 304, and (**e**) steel composite samples.

Visible pitting of samples was observed following the 144-h or 288-h treatment in ferric chloride solution. Typical corrosion pitting for each stainless steel type and the composite samples is shown in Figure 6. Type 430 had the most visible pitting at 144 and 288 h with frequent, long, and shallow pits along the length of the gage. The extent of pitting of the Type 430 samples was the greatest compared to other steel types. Of the stainless steel plates, Type 316 exhibited the least amount of visible pitting with small, circular pits scattered around the samples. Type 17-4 and 304 showed pits similar in appearance to, but more severe than, those present in Type 316. Pitting was more frequent around the edges of Type 17-4 and 304 and some pits formed holes through the thickness of these specimens. Composite samples showed little to no visible corrosion damage. Discoloration in the resin around the cross yarns was observed.

Table 2 indicates how much weight was retained by each sample after being subjected to the corrosion bath. Type 430 lost the most weight after corrosion. The specimens on average retained 91.8% of their weight after submersion for 144 h, and 84.6% of their weight after submersion for 288 h. On average, Type 17-4 retained 97.2% of original weight after 144 h, and 96.5% of original weight after 288 h. Type 316 samples had the highest average weight retained for the steel coupons after corrosion, retaining an average of 99% and 99.5% of their original weights after 144 and 288 h, respectively. Weight loss between 144 and 288 h did not change significantly in Type 316. Type 304 performed similarly to Type 17-4, and retained an average of 96.7% of original weight after 144 h and 95.2% of original weight after 288 h. The composite samples retained the most weight of all specimens on average when tested after 144 h, retaining 99.15% of original weight. On average, composite samples retained 95.44% of their original weight after 288 h, indicating that weight loss of composite samples was slightly higher than for Type 316 samples after 288 h of corrosion. It is not clear if this increase is statistically significant, however. The vinyl ester dog bones corroded for 144 h retained 100% of their original weight.

### 3.2. Tensile Experiment

Results of the tensile testing experiments are summarized in Table 2. For each sample type, the maximum stress and maximum strain are included. Percent strength lost indicates how much strength each sample lost when comparing it to the average failure stress of the control specimens of its type. Similarly, percent strain lost indicates how much strain to failure a specimen lost when comparing it to the minimum failure strain of the control specimens of its type. The number of specimens that failed outside of the extensometer gage length for each type is also included in Table 2.

**Figure 7:**
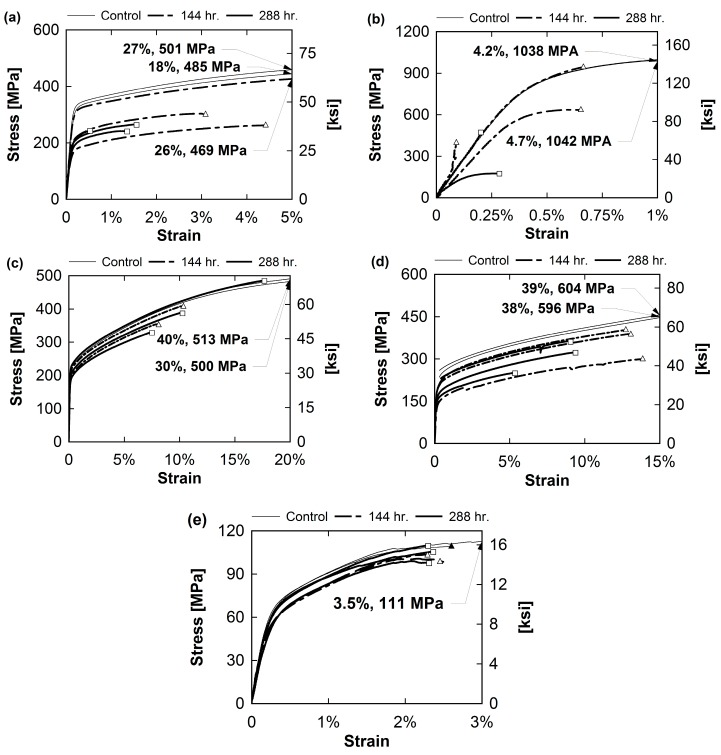
Stress–strain curves for stainless steel (**a**) Type 430, (**b**) Type 17-4, (**c**) Type 316, (**d**) Type 304, and (**e**) stainless steel fiber composites. Symbols on each line indicate when the sample failed.

Stress-strain curves for each sample type are shown in Figure 7. Symbols on each line indicate when the sample failed. If the failure location is not visible due to strain axis limitations for a particular sample, an arrow has been inserted to indicate the failure stress and failure strain.

Type 430 had an average maximum stress of 485 MPa (70.34 ksi) and average maximum strain of 23.6% in control specimens. These averages are greatly reduced in the 144- and 288-h Type 430 samples. 144-h samples had an average maximum stress of 277 MPa (40.25 ksi) and maximum strain of 2.7%. 288-h samples had an average maximum stress of 250 MPa (36.25 ksi) and a 1.1% average maximum strain. Type 17-4 samples had the highest initial strength with an average maximum stress of 1040 MPa (150.84 ksi) in control samples. The control samples had an average maximum strain of 4.5%. The average maximum strain was greatly reduced in 144- and 288-h samples, which had an average maximum strength of 659 MPa (95.63 ksi) and 324 MPa (47 ksi), respectively. The average maximum strain of the 144-h samples was 0.5% and the average maximum strain of the 288-h samples was 0.2%. Control specimens of Type 316 stainless steel had an average maximum stress of 506 MPa (73.4 ksi) and average maximum strain of 35.2%. 144-h samples had an average maximum stress of 384 MPa (55.7 ksi) and average maximum strain of 9.6%. The 288-h samples had an average maximum stress of 408 MPa (59.2 ksi) and average maximum strain of 13.1%. Type 304 had strengths similar to Type 316. The control specimens had an average strength of 600 MPa (87 ksi) and an average maximum strain of 38.6%. The 144-h samples had an average maximum strength of 363 MPa (52.6 ksi) and maximum strain of 13.2% while the 288-h samples had an average maximum stress of 397 MPa (57.6 ksi) and maximum strain of 9.3%. Lastly, the composite samples had an average maximum strength of 110 MPa (15.6 ksi) and maximum strain of 3.1% in control specimens. The 144-h samples had an average maximum strength of 94 MPa (13.6 ksi) and strain of 2.4%. The 288-h samples had an average maximum strength of 104 MPa (151.8 ksi) and maximum strain of 2.3%. The vinyl ester dog bones corroded for 144 h had an average maximum strength of 38.6 MPa (5.6 ksi) and maximum strain of 1.36%.

## 4. Discussion

**Figure 8:**
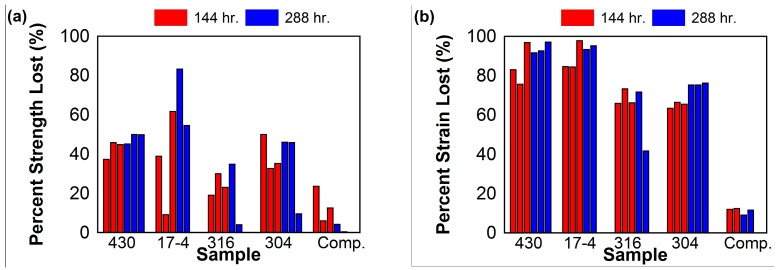
(**a**) Percent strength lost and (**b**) percent strain lost of each corroded sample when compared to their respective control specimens.

**Figure 9:**
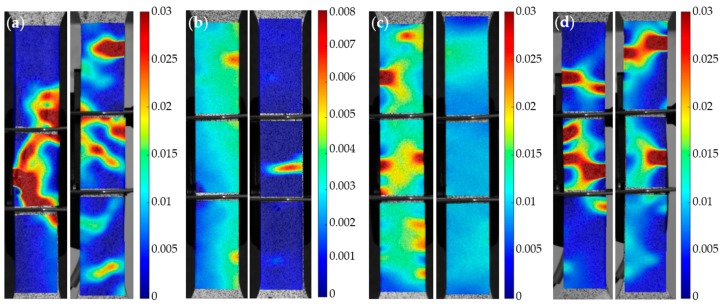
Longitudinal Eulerian strain concentrations for a selected 144- and 288-h sample of (**a**) Type 430, (**b**) Type 17-4, (**c**) Type 316, and (**d**) Type 304 stainless steel at a global strain of 1.5%.

**Figure 10:**
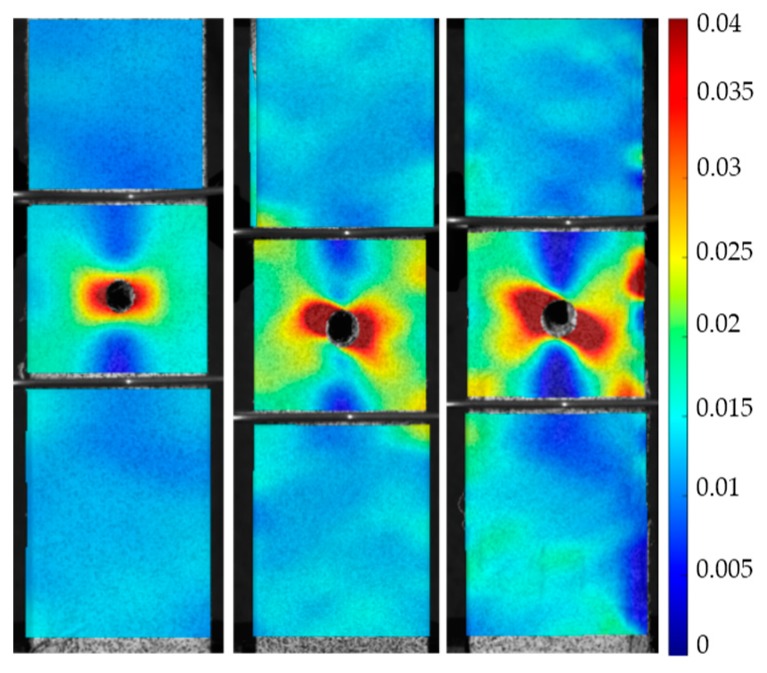
Longitudinal Eulerian strain concentrations for a selected control, 144-h, and 288-h composite sample at a global strain of 1.5%.

Percent loss of maximum strength and maximum strain for each corroded sample is visualized in the bar graphs shown in Figure 8a,b, respectively. 2D digital image correlation (DIC) was performed using NCorr, an open source 2D-DIC Matlab software [59]. Longitudinal Eulerian strain contours for selected 144- and 288-h Type 430, 17-4, 316, and 304 stainless steel samples are shown in Figure 9. These contours consist of the strains that were distributed when global strain was 1.5%. Longitudinal Eulerian strain contours for selected control, 144-h, and 288-h composite samples are shown in Figure 10. These contours consist of the strains that were distributed when global strain was also 1.5%.

Typical chemical composition of Type 430 stainless steel would leave it the most vulnerable to pitting corrosion in comparison to the chromium-nickel stainless steel types included in this study. This was reflected in the consistent pitting of samples, as well as the large percentages of maximum stress and maximum strain lost following corrosion. On average, 42.7% of strength was lost in the 144-h samples and 48.4% of strength was lost in the 288-h samples. Localized failure in the pits of the corroded Type 430 samples caused failure strains to be greatly reduced. On average, 85.1% of the maximum strain was lost in the 144-h samples and 93.7% of the maximum strain was lost in the 288-h samples. The localized failure due to larger pits is visualized in Figure 9a.

Type 17-4 had similar loss in failure strain due to localized failure in pits. The 144-h 17-4 samples had an average 88.9% reduction in maximum strain, and the 288-h Type 17-4 samples had an average 94.3% reduction in maximum strain. Loss of strength, however, varied more in the Type 17-4 specimens. On average, the 144-h samples had a 36.6% loss in strength, and the 288-h samples had a 68.9% loss in strength. As in Type 430, failure in Type 17-4 samples occurred around particularly large pits (Figure 9b).

Type 304 performed slightly better than stainless steel Types 430 and 17-4 when considering mechanical properties. On average, the 144-h Type 304 samples had a reduction in strength of 39.3% and reduction in strain of 65.1%. The 288-h samples had an average reduction in strength of 33.8% and reduction in strain of 75.6%. The strain distribution of the Type 304 samples indicates high strain concentrations around many pits (Figure 9d).

Of the stainless steel plate samples, Type 316 was the least vulnerable to corrosion and loss of strength. This result is expected, as the composition of Type 316 prevents extensive corrosion. Type 316 had a similar reduction in strain to Type 304. On average, the 144-h samples had a reduction in strain of 68.4%, and the 288-h samples had an average reduction in strain of 56.7%. Of the stainless steel coupons, Type 316 retained the most of its strength. 144-h samples had an average of 24.1% strength lost, and 288-h samples had an average 19.5% of strength lost. Much like Type 304, Type 316 had high strain distributions around more than one pit before failure, but the 288-h sample had the lowest strain distribution at 1.5% global strain (Figure 9c).

The corroded SFRP samples retained the most strength and strain to failure. The 144-h samples had an average reduction in strength of 14.9% and reduced strain of 5.2%. The 288-h samples had an average 5.8% reduction in strength and 11% loss in strain. An increase in strain concentrations around the hole can be seen in the corroded samples when comparing them to the control (Figure 10). It is important to note that the vinyl ester dog bones retained 100% of their strength after being corroded for 144 h and retained 90% of their maximum strain. This demonstrates that the resin contributes to the material durability of the composites when exposed to harsh environments.

The stainless steel fibers had been previously studied using a different matrix. McBride et al. used an EPON 828 liquid epoxy resin with an EPIKURE 3055 hardener to manufacture an 8-layer unidirectional steel fiber composite using a hand layup technique [1,2]. Following an open-hole tensile test, the stress–strain properties of the composites were derived to have an ultimate tensile strength of 161 MPa and an ultimate strain of 4.50%. The matrix properties were derived to have an ultimate tensile strength of 56.9 MPa and failure strain of 5.06%, so it is understandable that the mechanical properties of this composite are higher than that of those found in this study.

## 5. Conclusions

Type 430 stainless steel coupons proved to be the most susceptible to pitting corrosion, which was expected of this general-purpose type of steel due to its chemical composition. These corrosion samples had great loss in strength and strain, as well as the largest weight loss. Pitting was also most prevalent on these samples. Type 17-4 performed better than Type 430 in mechanical testing performance and weight loss after corrosion, but was shown to be one of the more susceptible types of stainless steel tested. Type 304 had weight loss similar to Type 17-4, but was able to retain more of its strength and strain to failure compared to Type 17-4. Of the stainless steel plates, Type 316 had the best weight retention and was able to retain the most strength. Overall, the composite samples were the least susceptible to pitting corrosion. They were able to retain the most strength and strain. The weight loss of the composite samples was similar to the weight loss of Type 316 samples, Type 316 having retained slightly more weight after corrosion. The findings of this study further promote that SFRP composites are a viable alternative to conventional metals for corrosion resistance.

## 6. Future Work

Additional studies need to be performed in order to completely understand hybrid composites and corrosion effects. The interfacial fiber–matrix properties are of critical importance to achieving desirable composite material performance. To improve the toughness of the composite, one can study the effect corrosion has on the fiber–matrix interfacial shear strength (IFSS) by determining the debonding force between micro-droplets of matrix on single reinforcement fibers under different corrosion conditions. Additionally, mechanical testing should be performed on corroded and noncorroded single fibers to obtain the mechanical properties. To completely understand the materials used in the study, elemental composition of the materials can be identified using energy dispersive X-ray analysis (EDX). Resistance to wear and environmental and corrosion conditions needs to be further studied by using a wear test.

## Figures and Tables

**Table 1:** Steel fiber chemical composition.

Fe	Cr	Ni	Mo	Mn	Si	Cu	V	Co
65.4%	20.2%	10.5%	2.4%	0.57%	0.49%	0.17%	0.14%	0.10%

**Table 2:** Percent of original weight, maximum stress, maximum strain, percent strength lost, and percent strain lost for each sample. Number of failures outside the gage length for each sample type is also included.

Sample *	% of Original Weight	Maximum Stress MPa (ksi)	Maximum Strain (%)	Percent Strength Lost (%)	Percent Strain Lost (%)	No. Failures Outside Gage Length
430-0-1	-	485 (70.3)	18.2	-	-	0
430-0-2	-	501 (72.6)	27.2	-	-	
430-0-3	-	469 (68.0)	25.6	-	-	
430-144-1	91.5	304 (44.1)	3.1	37.3	83.0	1
430-144-2	92.9	262 (38.0)	4.4	45.9	75.7	
430-144-3	90.8	267 (38.7)	0.6	44.9	96.8	
430-288-1	84.3	265 (38.5)	1.5	45.2	91.6	2
430-288-2	86.2	242 (35.1)	1.4	50.0	92.6	
430-288-3	83.3	243 (35.2)	0.5	49.9	97.1	
17-4-0-1	-	1038 (150.6)	4.2	-	-	0
17-4-0-2	-	1042 (151.2)	4.7	-	-	
17-4-144-1	97.2	636 (92.2)	0.7	38.9	84.6	2
17-4-144-2	97.6	944 (137.0)	0.7	9.2	84.4	
17-4-144-3	96.7	398 (57.7)	0.1	61.7	97.8	
17-4-288-1	96.6	175 (25.3)	0.3	83.2	93.3	0
17-4-288-2	96.3	473 (68.5)	0.2	54.6	95.2	
316-0-1	-	500 (72.6)	30.3	-	-	0
316-0-3	-	513 (74.4)	40.1	-	-	
316-144-1	99.3	410 (59.4)	10.3	19.1	65.9	2
316-144-2	98.8	354 (51.4)	8.1	30.0	73.3	
316-144-3	99.0	389 (56.5)	10.3	23.1	66.2	
316-288-1	99.4	330 (47.9)	8.6	34.8	71.7	2
316-288-2	99.6	486 (70.4)	17.7	4.1	41.7	
304-0-1	-	596 (86.4)	37.9	-	-	0
304-0-2	-	604 (87.6)	39.3	-	-	
304-144-1	96.2	300 (43.5)	13.9	50.0	63.4	2
304-144-2	96.8	403 (58.5)	12.7	32.7	66.4	
304-144-3	97.1	388 (56.3)	13.1	35.2	65.5	
304-288-1	93.6	324 (46.9)	9.4	46.1	75.3	2
304-288-2	95.0	325 (47.1)	9.4	45.9	75.3	
304-288-3	97.1	542 (78.7)	9.0	9.6	76.2	
Comp.-0-1	-	111 (16.2)	3.5	-	-	0
Comp.-0-2	-	109 (15.8)	2.6	-	-	
Comp.-144-1	98.87	84 (12.2)	2.6	23.6	−1.5	0
Comp.-144-2	99.43	104 (15.0)	2.3	6.1	12.0	
Comp.-288-1	93.07	96 (14.0)	2.3	12.6	12.4	0
Comp.-288-2	96.75	106 (15.3)	2.4	4.3	9.1	
Comp.-288-3	96.50	110 (15.9)	2.3	0.5	11.6	

* Naming convention of each sample starts with the stainless steel type, followed by a 0, 144, or 288 for the control (0 h of corrosion), 144-h, and 288-h specimens, respectively. Numbers 1, 2, 3, or 4 at the end indicate a different sample of the same type.
